# Involvement of anterior and posterior corneal surface area imbalance in the pathological change of keratoconus

**DOI:** 10.1038/s41598-018-33490-z

**Published:** 2018-10-09

**Authors:** Koji Kitazawa, Motohiro Itoi, Isao Yokota, Koichi Wakimasu, Yuko Cho, Yo Nakamura, Osamu Hieda, Shigeru Kinoshita, Chie Sotozono

**Affiliations:** 1 Department of Ophthalmology, Kyoto Prefectural University of Medicine, Kyoto, Japan; 2 Department of Frontier Medical Science and Technology for Ophthalmology, Kyoto Prefectural University of Medicine, Kyoto, Japan; 3 Baptist Eye Institute, Kyoto, Japan; 4 Department of Biostatistics, Kyoto Prefectural University of Medicine, Kyoto, Japan

## Abstract

Keratoconus (KC) is an ectatic disorder with a high prevalence rate. However, the exact cause of the disease and possible underlying mechanisms of development remain unclear. In this present study, we aimed to investigate the anterior and the posterior corneal surface area in normal, forme fruste keratoconus (FFKC), and keratoconic eyes (as a reference group) using anterior segment optical coherence tomography (AS-OCT) in order to assess the pathological change of KC. The surface areas of the anterior or posterior cornea, and the anterior-posterior (As/Ps) ratio of corneal surface area, were measured at the central 5.0 mm-, 6.0 mm-, and 7.0 mm-diameter areas via AS-OCT, and a comparison between the normal eyes and FFKC eyes was then performed using the Mann-Whitney U test. The posterior surface area at the central 5.0 mm areas in the FFKC eyes (20.430 mm^2^) and KC eyes (20.917 mm^2^) seemed to become larger than that of normal eyes (20.389 mm^2^) (normal vs FFKC; *P* = 0.06). Moreover, the As/Ps of the corneal surface area in the FFKC eyes (0.986) and the KC eyes (0.976) was significantly smaller than that of the normal eyes (0.988) (normal vs FFKC; *P* < 0.01). Anterior and posterior corneal surface area imbalance may reflect keratoconic eyes at the early stage of the disease.

## Introduction

It has been reported that keratoconus (KC) is usually a bilateral^1,2^ and asymmetric^3,4^ ectatic disorder characterized by corneal protrusion, thus leading to irregular astigmatism^5^. The reported incidence of KC is estimated to be between 50–230 per 100,000, with a prevalence of 54.5 per 100,000 in the general population^2,6^.

The development of various technologies now allows for the anterior and posterior shape of the cornea to be analyzed in detail^5,7–12^. Although recent studies have reported the relevance of the posterior corneal curvature in the screening and diagnosis of KC^7,8^, there is an ongoing debate as to whether the first signs of KC at the initial stage of the disease appear at the corneal anterior surface^9^, posterior surface^5,10^, or both^11^. It has been reported that the development of keratoconus involves intralamellar displacement and slippage that leads to thinning of the central cornea and associated corneal curvature changes^13,14^ and it is caused by genetic factors^15,16^, increased oxidative stress^17–19^, higher matrix metalloproteinase levels^20,21^, and hormones^22,23^. However, the exact cause of the disease and possible underlying mechanisms of development remain unclear.

In 1961, Amsler^24^ described forme fruste keratoconus (FFKC) as ‘an incomplete, abortive, or unusual form of a syndrome or disease’, and it is reportedly defined as the contralateral eye with a normal shape in cases of unilateral KC^25–27^. The findings in a recent study showed that in 50% of the subjects with unilateral KC, the non-affected fellow eye will develop the disease within 16 years^28^. Thus, FFKC eyes may provide an insight into the early stage of KC^29^.

We previously reported a formula to calculate the precise corneal surface area using anterior segment optical coherence tomography (AS-OCT)^30^. However, and to the best of our knowledge, there have been no previous studies to investigate the corneal surface area in keratoconic eyes. The purpose of this present study was to investigate the three-dimensional (3D) corneal shape in eyes with early-stage KC, and analyze and compare the anterior and posterior surfaces and the ratio of these surface areas in normal, FFKC, and KC eyes via the use of AS-OCT.

## Results

### Demographic data

**Figure 1:**
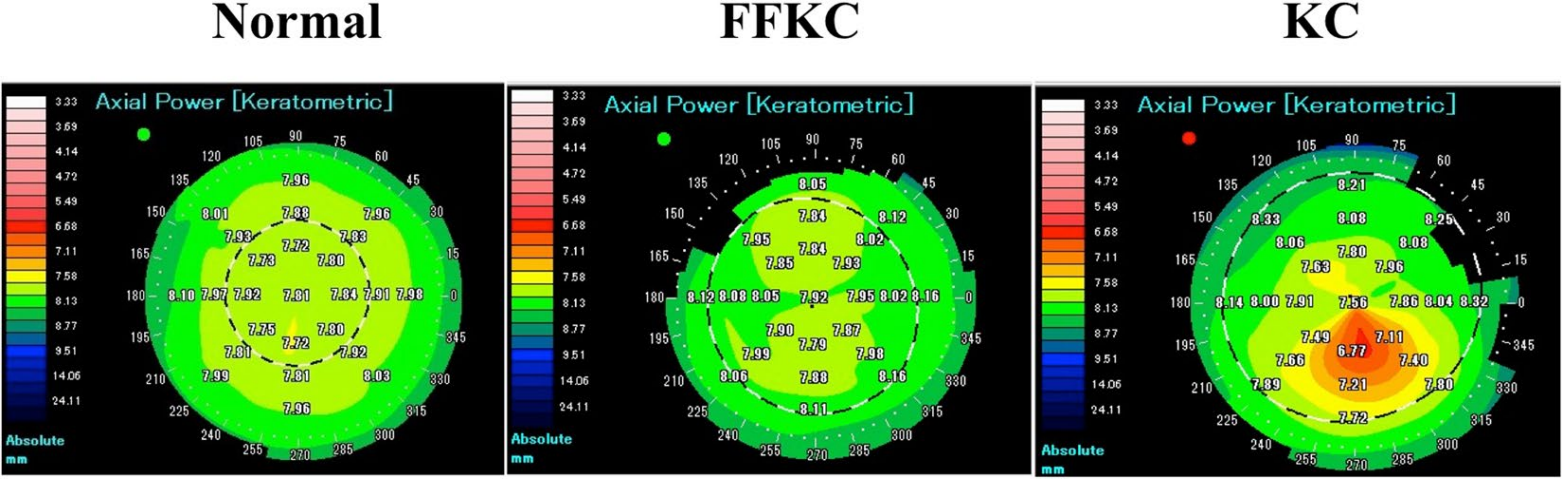
Front axial power/curvature map in anterior segment optical coherence tomography (OCT) images. Left-side image: normal cornea, middle image: forme fruste keratoconus (FFKC) cornea, right-side image: keratoconus (KC) cornea.

This study involved 14 eyes of 14 FFKC patients, 23 eyes of 23 KC patients, and 25 eyes of 25 normal healthy subjects (the demographic data is shown in Table 1). The KC eyes were used as the reference group for analysis, and the normal-group control eyes were adjusted with subject age. The severity of KC patients was classified into 4 groups according to the Amsler-Krumeich classification for grading keratoconus^31^; Grade 1 (7 eyes), Grade 2 (4 eyes), Grade 3 (6 eyes), and Grade 4 (6 eyes). The corneas with central and paracentral cones according to the corneal thickness and corneal topography were 5 eyes and 9 eyes in FFKC patients, and 12 eyes and 11 eyes in KC patients, respectively. No significant difference in best spectacle corrected visual acuity (BSCVA) was found between the normal control eyes and the FFKC eyes (Table 1). Three representative cases are shown in Fig. 1.

**Table 1:** Demographic data.

	Total (n = 62)	Normal (n = 25)	FFKC (n = 14)	KC (n = 23)	N vs. FFKC (*P* value)
Median Age (years)	29.0	32.0	29.5	27.0	0.85
Age Range (years)	16–39	16–36	20–39	20–39	
Male (%)	75.8	64.0	78.6	90.0	0.36
Median BSCVA (logMAR)	−0.08	−0.18	−0.08	0.22	0.07
BSCVA Range	−0.30–1.40	−0.30–0.00	−0.30–0.00	−0.18–1.40	

FFKC: forme fruste keratoconus, KC: keratoconus, N: Normal, BSCVA: best spectacle-corrected visual acuity, logMAR; logarithm of Minimum Angle of Resolution.

### Intergroup differences of AS-OCT parameters

**Figure 2:**
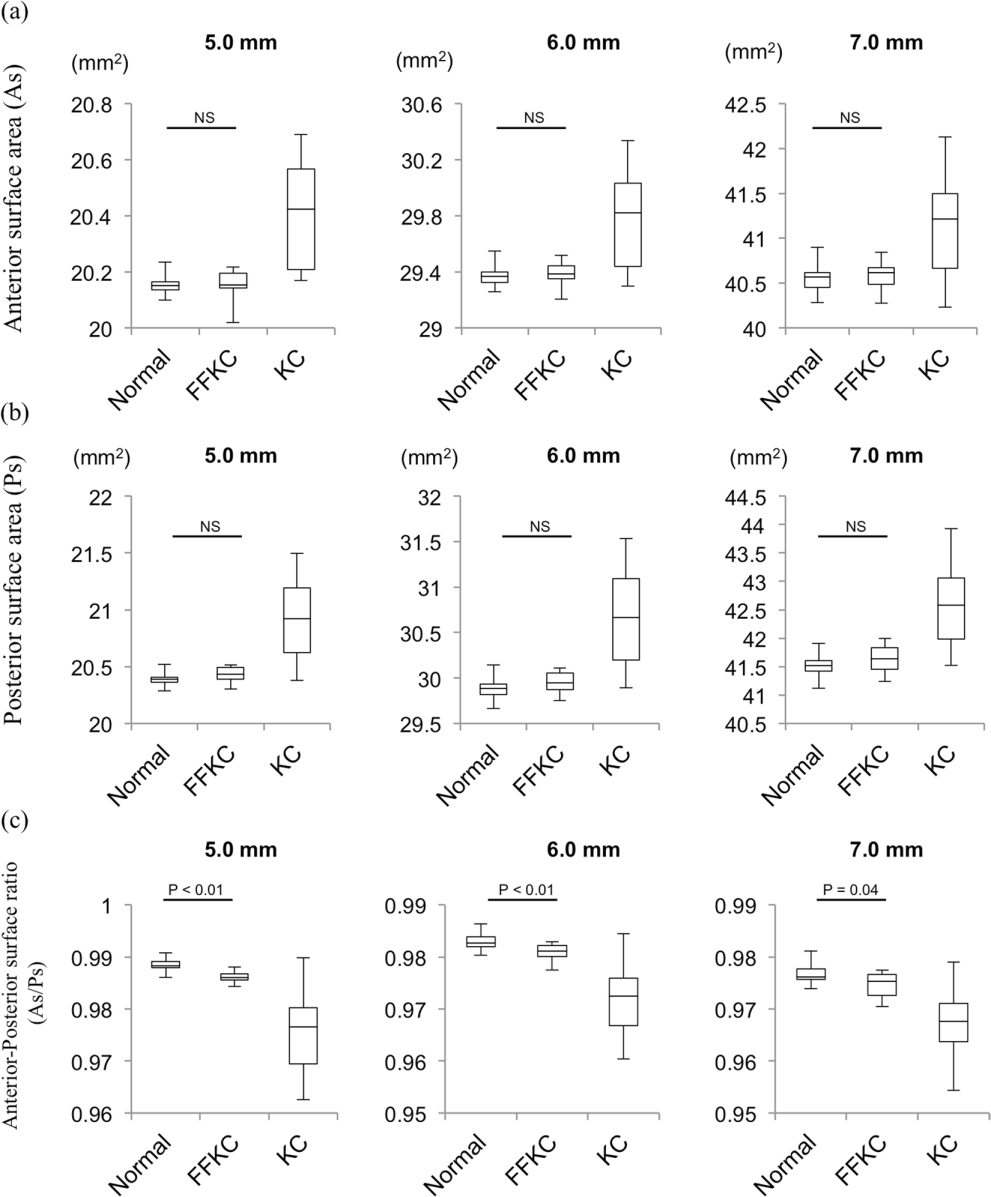
Anterior surface area (As); (**a**) posterior surface area (Ps); (**b**) and anterior-posterior surface ratio (As/Ps); (**c**) in normal, forme fruste keratoconus (FFKC), and keratoconus (KC) subjects. Left-side column: the central 5.0-mm diameter area, Middle column: the central 6.0-mm diameter area, Right-side column: the central 7.0-mm diameter area. As/Ps was significantly smaller in the FFKC eyes than in the normal eyes at the central 5.0-mm, 6.0-mm, and 7.0-mm measurement point areas. NS: not significant.

**Figure 3:**
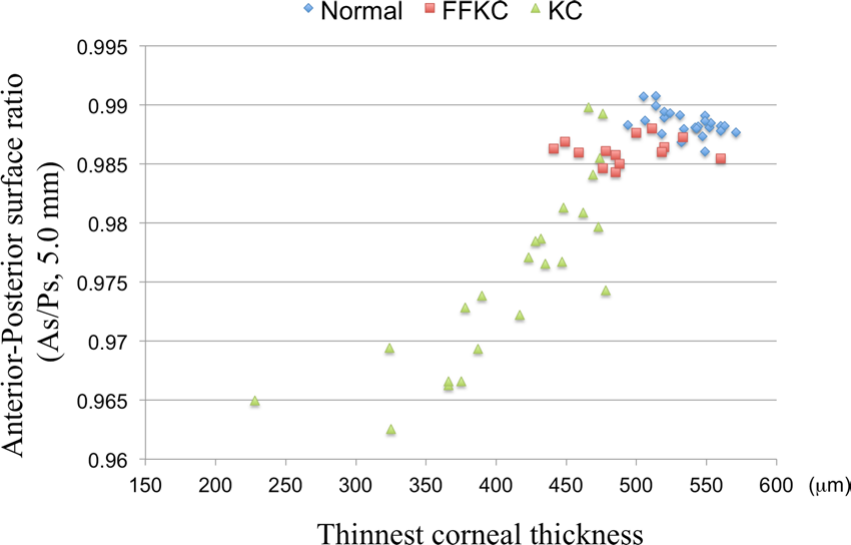
Graph illustrating the correlation between anterior-posterior surface ratio (As/Ps) and the thinnest corneal thickness. A positive correlation was found between the As/Ps and the thinnest corneal thickness (r = 0.879, *P* < 0.001). FFKC: forme fruste keratoconus, KC: keratoconus.

The AS-OCT parameters, including keratometry, pachymetry, and anterior and posterior ‘best-fit sphere’ (BFS) radius values, for the different areas in the normal control, FFKC, and KC eyes are shown in Table 2. The pachymetric parameters (i.e., the central corneal thickness and the thinnest corneal thickness) were found to be significantly different between the normal-group eyes and the FFKC-group eyes (*P* < 0.01 and *P* < 0.01, respectively) (Table 2). No significant differences in anterior and posterior BFS was found between the normal and FFKC eyes, however, a significant difference in the anterior-posterior ratio of BFS (Ab/Pb) radius values was found between the normal and FFKC eyes at the central 5.0-, 6.0-, and 7.0-mm diameter areas. Moreover, no significant difference in the anterior and posterior surface areas was found between the normal and FFKC eyes. However, a significant difference in the anterior-posterior ratio of the corneal surface (As/Ps) was found at the central 5.0-, 6.0-, and 7.0-mm diameter areas. The As/Ps at the 5.0-mm to 7.0-mm diameter area was smaller in the FFKC and KC eyes than in the normal eyes (Table 3 and Fig. 2). A significant correlation was found between As/Ps at the central 5-mm area and the thinnest corneal thickness (r = 0.879, *P* < 0.001) (Fig. 3). The ROC-AUC of As/Ps at the 5.0-mm was 0.946, while, the ROC-AUCs of central corneal thickness and thinnest corneal thickness were 0.829 and 0.859, respectively.

**Table 2:** The anterior segment optical coherence tomography (AS-OCT) parameters in normal, forme fruste keratoconus (FFKC), and keratoconic eyes.

		Normal	FFKC	KC	N vs. FFKC
		(n = 25)	(n = 14)	(n = 23)	(*P*-value)
Keratometry	Ks (D)	43.41	43.65	54.62	0.36
	Kf (D)	42.28	42.00	50.50	0.86
	Avg. K (D)	42.84	42.83	52.56	0.60
Pachymetry	Center (μm)	539.76	501.71	436.22	<0.01
	Thinnest (μm)	535.76	493.07	411.61	<0.01
Anterior BFS	5 mm (mm)	7.88	7.89	6.41	0.58
	6 mm (mm)	7.90	7.90	6.61	0.49
	7 mm (mm)	7.92	7.91	6.80	0.40
Posterior BFS	5 mm (mm)	6.61	6.51	5.03	0.28
	6 mm (mm)	6.62	6.53	5.28	0.21
	7 mm (mm)	6.65	6.56	5.52	0.27

FFKC: forme fruste keratoconus, KC: keratoconus, N: Normal, Ks: steep keratometry, Kf: flat keratometry, Avg. K: average keratometry, BFS: best-fit sphere.

**Table 3:** Anterior-posterior surface area in normal, forme fruste keratoconus (FFKC), and keratoconic eyes.

		Normal	FFKC	KC	N vs. FFKC
		(n = 25)	(n = 14)	(n = 23)	(*P*-value)
Anterior Surface area	5 mm (mm^2^)	20.154	20.147	20.403	0.62
	6 mm (mm^2^)	29.370	29.380	29.796	0.41
	7 mm (mm^2^)	40.545	40.579	41.194	0.33
Posterior Surface area	5 mm (mm^2^)	20.389	20.430	20.917	0.06
	6 mm (mm^2^)	29.881	29.953	30.662	0.07
	7 mm (mm^2^)	41.513	41.642	42.592	0.13
As/Ps	5 mm	0.988	0.986	0.976	<0.01
	6 mm	0.983	0.981	0.972	<0.01
	7 mm	0.977	0.974	0.967	0.04
Ab/Pb	5 mm	1.192	1.216	1.284	0.01
	6 mm	1.196	1.218	1.329	0.01
	7 mm	1.196	1.214	1.297	0.02

FFKC: forme fruste keratoconus, KC: keratoconus, N: Normal, VA: visual acuity, As: anterior surface area, Ps: posterior surface area, Ab: anterior best-fit sphere, Pb: posterior best-fit sphere.

### Corneal curvature and surface area

In order to determine whether Ab/Pb or As/Ps is the optimal index to detect early-stage KC, the ‘receiver operating characteristic area under curve’ (ROC-AUC) was used to calculate the corneal curvature and surface area. At the central 5.0-, 6.0-, and 7.0-mm areas, ROC-AUC using Ab/Pb to separate normal from FFKC eyes was 0.742, 0.751, and 0.729, respectively, while ROC-AUC using As/Ps analysis to separate normal from FFKC eyes was 0.948, 0.797, and 0.732, respectively, thus revealing higher ROC-AUCs at each measurement point compared with when Ab/Pb was used (Table 4). Furthermore, the ROC-AUCs of As/Ps and Ab/Pb at the 5.0-mm area in FFKC patients with the central cone pattern was 0.984 and 0.896, respectively, and the ROC-AUCs of As/Ps and Ab/Pb in FFKC patients with the paracentral cone pattern was 0.924 and 0.587, respectively.

**Table 4:** The receiver operating characteristic area under curve (ROC-AUC) in each parameter to separate normal eyes from forme fruste keratoconus (FFKC) eyes.

		ROC-AUC	95% CI
Anterior Surface area	5 mm	0.449	0.226–0.672
	6 mm	0.577	0.364–0.790
	7 mm	0.586	0.377–0.795
Posterior Surface area	5 mm	0.678	0.467–0.890
	6 mm	0.672	0.472–0.873
	7 mm	0.655	0.454–0.857
As/Ps	5 mm	0.948	0.882–1.000
	6 mm	0.797	0.645–0.948
	7 mm	0.732	0.550–0.915
Ab/Pb	5 mm	0.742	0.552–0.931
	6 mm	0.751	0.569–0.933
	7 mm	0.729	0.546–0.913

ROC-AUC: the receiver operating characteristic area under curve, CI: confidence interval, As: anterior surface area, Ps: posterior surface area, Ab: anterior best-fit sphere, Pb: posterior best-fit sphere.

## Discussion

In this present study, we were able to successfully demonstrate corneal anterior and posterior surface areas via examination by AS-OCT, a non-contact and non-invasive 3D imaging system. The As/Ps (i.e., the ratio of corneal anterior and posterior surface areas) was significantly smaller in FFKC and KC eyes than in normal healthy eyes, and the posterior surface area became relative large in comparison with the anterior surface area in FFKC and KC eyes. Furthermore, when using As/Ps at the central 5.0-mm diameter area to separate FFKC eyes from normal eyes, ROC-AUC was found to be 0.946, much higher than the ROC-AUC of 0.742, 0.829, and 0.859 when using Ab/Pb (i.e., the ratio of corneal anterior and posterior BFS), central corneal thickness, and thinnest corneal thickness, respectively, thus suggesting that As/Ps is a more reliable index to detect keratoconic eyes at the early stage of the disease because As/Ps estimates the precise captured corneal shape with a 3D structure.

In this present study, our findings revealed that the As/Ps ratio was smaller in FFKC and KC eyes, thus suggesting, theoretically, that the posterior surface area became enlarged compared to the anterior surface at the initial stage of the disease. However, it still remains unclear as to whether changes in the anterior surface^9^, posterior surface^5,10^, or both surfaces of the cornea^11^ are the first to arise in the early stage of KC. In keratoconic eyes, the organization of the collagen lamellae is disrupted, and the distribution of microfibril bundles is very different, thus resulting in a loss of tensile strength and the progression of ectasia^13,32^. Our findings, which demonstrated the smaller As/Ps ratio in keratoconic eyes via 3D analysis, suggest that a protrusion of the posterior cornea occurs prior to changes being observed on the anterior corneal surface, thus leading to enlargement of the posterior corneal surface area compared to the anterior surface area and the formation of early-stage KC.

Moreover, our findings revealed that the As/Ps ratio represented an imbalance of the anterior and posterior cornea. Interestingly, we found a significant difference in the anterior-posterior ratio of BFS (i.e., Ab/Pb), as well as in the ratio of corneal anterior and posterior surface areas (i.e., As/Ps), between the normal and FFKC eyes, although keratometric values, including BFS, were considered to be insufficient for detecting FFKC^27,33^. Further analysis of the corneal surface area revealed that ROC-AUC at the central 5.0-mm to 7.0-mm areas was higher when using As/Ps than when using Ab/Pb. This discrepancy might possibly be explained by the difference between two-dimensional (2D) systems and 3D systems. As we observed in our previous report^30^, AS-OCT provided precise corneal curvature at each measurement point based on elevation map, and calculated the corneal surface area by multiplying the corneal curvature. This suggests that the precise corneal shape was capable of being captured with a 3D structure, thus resulting in the high ROC-AUC (0.948) to distinguish FFKC from normal eyes.

In addition, our findings revealed that the As/Ps ratio might represent the corneal volume as well, because there was a significant positive correlation between As/Ps at the central 5-mm diameter area and the thinnest corneal thickness (r = 0.879, *P* < 0.001). In fact, FFKC and KC eyes showed not only lower As/Ps, but also thinner corneal thickness. It should be noted that corneal thickness is known to be the least reliable indicator for diagnosing KC^5^. Thus, the As/Ps ratio can be a novel potential index to illustrate not only the corneal shape, but also the corneal volume.

It should be noted that this study did have several limitations. First, it was a retrospective study. However, the subjects were age matched and adjusted to precisely evaluate the findings. In order to conduct a more definitive analysis, a prospective study should be performed. Second, the sample size in this present study was small. Thus, a larger sample size is needed to confirm our findings.

In conclusion, our findings show that the corneal surface area can be calculated by using AS-OCT and that the anterior-posterior ratio of the corneal surface can illustrate the corneal shape and volume as a 3D structure, thus revealing that an imbalance of the anterior-posterior ratio of the corneal surface can reflect the initial appearance of KC.

## Methods

### Subjects

This retrospective study involved subjects seen at the Baptist Eye Institute, Kyoto, Japan between 2015 and 2017. The study was performed in accordance with the tenets set forth in the Declaration of Helsinki, and was approved by the Kyoto Ethics Review Committee, Kyoto, Japan (Approval #1604); an independent organization established to approve ethical issues. Written informed consent was obtained from all subjects prior to their participation in the study. Clinical registration of this study was obtained from UMIN UMIN000024891 (http://www.umin.ac.jp/english/).

This study involved 62 eyes of 62 subjects seen at the Baptist Eye Institute and who were classified into one of the following three groups: 1) the normal group, 2) the FFKC group, and 3) the KC group according to corneal topography as determined by anterior eye segment tomography (Pentacam^**®**^ HR version 1.20; Oculus Optikgeräte GmbH, Wetzlar, Germany) and slit-lamp examination. The normal-group subjects were enrolled as an age-matched control to the FFKC-group subjects. FFKC was defined as the fellow eye in the unilateral KC cases who had a normal anterior topography map by Pentacam^**®**^ HR and slit-lamp examination, as previously reported^27^, thus indicating the early stage of KC. The KC-group eyes were used as a reference for analysis. The diagnosis of KC was based on anterior corneal topography mapping, such as focal or inferior steepening, a bow-tie pattern, and skewed axes^11^, as well as at least one of the clinical symptoms observed via slit-lamp examination; i.e., a Fleischer ring, Vogt’s striae, or corneal thinning. The severity of KC patients was classified into 4 groups according to Amsler-Krumeich classification for grading keratoconus^31^, based on mean keratometry on the anterior curvature sagittal map, thickness at the thinnest location, and the refractive error of the patient. In addition, FFKC and KC patients were separated into the central cone pattern or the paracentral cone pattern, according to the asymmetry of the corneal thickness and corneal topography. The eyes with no indications of KC were classified as normal eyes. If both eyes were diagnosed as normal, then one eye was randomly selected for further analysis. Patients with a history of ocular surgery, acute corneal hydrops, or other ocular diseases with refractive error were excluded from the study. To precisely evaluate the shape of the cornea, corneal topography was examined after contact lenses had been removed for at least 2 weeks (for soft contact lenses) or 4 weeks (for rigid contact lenses). Best spectacle-corrected visual acuity without wearing contact lenses was examined.

### Imaging Methods

In each examined eye, the anterior and posterior corneal surface was measured by swept-source AS-OCT (SS-1000 CASIA; Tomey Corporation, Nagoya, Japan), a non-invasive, non-contact, 3D imaging system with a center wavelength of 1310 nm and a scanning speed of 30,000 A lines/second. We confirmed the high reproducibility of each examination in corneas of various shapes (Supplementary Table S1), demonstrating that both standard deviation and standard error were less than 0.0005, and intraclass correlation coefficient (ICC) based on evaluating the measurement process (EMP) was 0.9997. The anterior and posterior corneal surface areas in the 5.0-, 6.0-, and 7.0-mm diameter areas were calculated based on the anterior and posterior elevation map, as previously reported^30^. Briefly, we used the formula S = 2 × PI × R (R − √R^2^ − (D/2)^2^, in which S = surface area, PI = ratio of a circle’s circumference (3.14), R = curvature, and D = corneal diameter, at each measurement point based on the elevation map which was calculated via AS-OCT.

### Statistical Analyses

Statistical analyses were performed using R version 3.1.0 (The R Foundation, https://www.r-project.org/foundation/) statistical software, with the data being presented as mean value or median value where applicable. Since the data were not normally distributed, the nonparametric Mann-Whitney U test was used to compare each parameter between the normal eyes and the FFKC eyes. A *P* value of <0.05 was considered statistically significant. Spearman’s correlation coefficient test was performed to analyze the correlation between As/Ps at the central 5.0-mm measurement point and the thinnest corneal thickness. ROC-AUC was calculated by the overall predictive accuracy of the test parameters.

## Electronic supplementary material

Supplementary Table S1
