# Supplementary figures and images for: Involvement of anterior and posterior corneal surface area imbalance in the pathological change of keratoconus
Source: Sci Rep. 2018 Oct 9;8:14993. doi: 10.1038/s41598-018-33490-z (PMC6177437; doi:10.1038/s41598-018-33490-z)

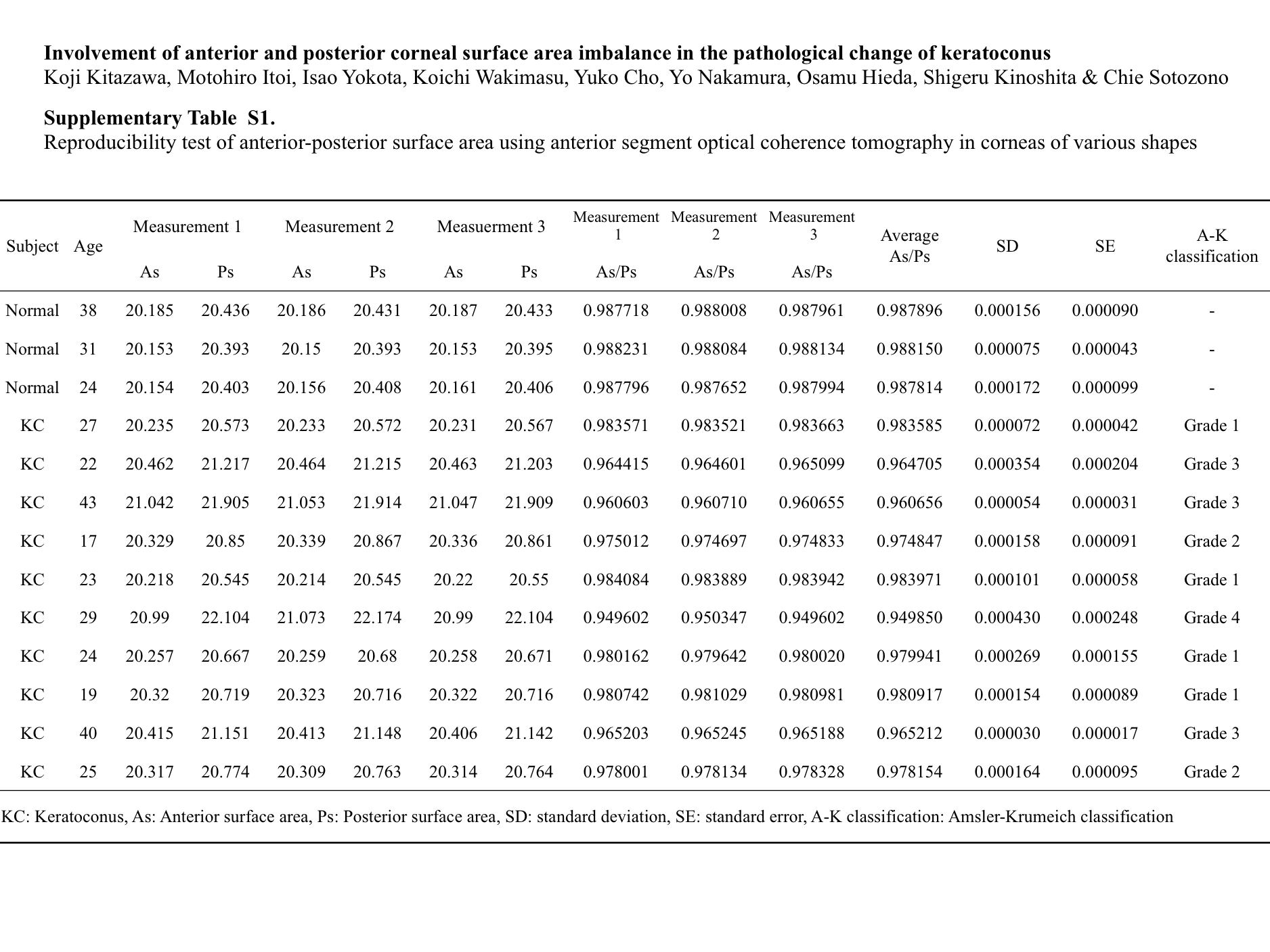

Supplement: Supplementary file 1 — Supplementary Table S1 [file 41598_2018_33490_MOESM1_ESM.tif]
